# Sex Determination Using Linear Anthropometric Measurements Relative to the Mandibular Reference Plane on CBCT 3D Images

**DOI:** 10.3390/jimaging11070224

**Published:** 2025-07-05

**Authors:** Nikolaos Christoloukas, Anastasia Mitsea, Leda Kovatsi, Christos Angelopoulos

**Affiliations:** 1 Department of Oral Diagnosis & Radiology, School of Dentistry, National and Kapodistrian University of Athens, 2 Thivon Str., 11527 Athens, Greece; 2 Laboratory of Forensic Medicine and Toxicology, School of Medicine, Aristotle University of Thessaloniki, 54124 Thessaloniki, Greece

**Keywords:** sex estimation, 3D reconstructed surfaces, CBCT, mandibular measurements

## Abstract

Sex determination is a fundamental component of forensic identification and medicolegal investigations. Several studies have investigated sexual dimorphism through mandibular osteometric measurements, including the position of anatomical foramina such as the mandibular and mental foramen (MF), reporting population-specific discrepancies. This study assessed the reliability and predictive ability of specific anthropometric mandibular measurements for sex estimation using three-dimensional (3D) cone beam computed tomography (CBCT) surface reconstructions. Methods: CBCT scans from 204 Greek individuals (18–70 years) were analyzed. Records were categorized by sex and age. Five linear measurements were traced on 3D reconstructions using ViewBox 4 software: projections of the inferior points of the right and left mental and mandibular foramina and the linear distance between mental foramina projections. A binary logistic regression (BLR) model was employed. All measurements showed statistically significant sex differences, with males presenting higher mean values. The final model achieved accuracy of 66.7% in sex prediction, with two vertical measurements—distances from the right mandibular foramen and the left mental foramen—identified as the strongest predictors of sex. The positions of the mandibular and mental foramina demonstrate sex-related dimorphism in this Greek sample, supporting their forensic relevance in population-specific applications.

## 1. Introduction

Certain characteristics, specifically sex, age, and ethnicity, can be used to define an individual. Accurate assessment of sex is considered a fundamental aspect of the human identification process [1].

The integrity of the available skeletal remains determines the accuracy of sex identification. If an entire adult skeleton is available for examination, sex can be predicted with approximately 100% accuracy [2]. Furthermore, in cases where a segmented skull is identified, the predictive ability decreases to 92%. In addition, when the entire skull is not available, the mandible plays a decisive role. The mandible is characterized by higher resistance to the taphonomic process than other skeletal structures [3].

The mandible exhibits sexual dimorphism from the earliest stages of life [4]. Specifically, previous studies have reported that sexual dimorphism is detectable as early as 5 months of age [5,6]. Moreover, mandibular sexual dimorphism decreases during childhood and adolescence (between 4 and 14 years of age) and increases during adulthood [7]. Among various mandibular morphological features, the mental foramen is considered one of the most robust landmarks [3].

Cone beam computed tomography (CBCT) is an advanced qualitative imaging method that provides unique visualization of hard dental tissues and bone structures [8]. Due to its high imaging speed and low radiation dosage, its effective radiation dosage is considerably lower than that of other 3D imaging modalities, such as multi-detector computed tomography (MDCT) [9]. In addition, the CBCT imaging system allows accurate examination of all mandibular aspects [10].

A limited number of published studies have investigated the accuracy of mandibular analysis regarding sex estimation in living individuals or skeletal remains of the Greek population. The majority of previous investigations assessed the shape and/or the size of the mandible, but none have estimated linear and/or angular measurements based on the accurate localization of specific anatomical landmarks or their projections [11,12,13]. The current study fulfills a gap in the forensic literature by focusing on linear measurements relative to a defined mandibular reference plane in a modern Greek sample. It provides novel, population-specific data that may contribute to developing sex estimation standards adapted to the Greek population.

The present study aimed to evaluate the reliability and predictive capacity of specific linear mandibular measurements for sex estimation on three-dimensional (3D) reconstructed surfaces derived from dental CBCT records of adult Greek individuals. These data were selected based on information retrieved from the electronic records of the School of Dentistry, National and Kapodistrian University of Athens. The database included information such as the individuals’ birthplace and surname, which were used as indicators of Hellenic ancestry. A secondary objective of this study included the development of a sex estimation formula with potential predictive accuracy.

## 2. Material and Methods

In our study, volumetric data obtained from 204 patients (50% males and 50% females), whose cone beam computed tomography (CBCT) imaging examinations were conducted at the School of Dentistry, National and Kapodistrian University of Athens (NKUA), were reprocessed and reconstructed. These imaging data were pre-existing records from the school’s database, initially acquired for diagnostic or treatment purposes unrelated to this study. To develop a predictive model with a binary categorical outcome (sex: male/female), targeting a small error (≤0.05) and 80% study power, a total sample size of 204 participants was required. The sample consisted of gender- and age-group-matched individuals (102 males and 102 females).

The study was also in accordance with the ethical standards of the Declaration of Helsinki and the guidelines of the Strengthening the Reporting of Observational Studies in Epidemiology (STROBE) statement [14]. Ethical approval was obtained from the Research and Ethics Committee of the School of Dentistry, National and Kapodistrian University of Athens, Greece (27 January 2025/677). Each patient’s CBCT volumetric data was anonymized before analysis, and only age and sex were collected. The approved protocol was followed without deviation from its original design.

The study sample was selected according to the following inclusion criteria: (a) the entire mandibular region was encompassed within the radiation field of view (FOV); (b) CBCT scans were performed exclusively using the NewTom VGI scanner (NewTom, Verona, Italy) at the School of Dentistry, Athens (NKUA); (c) appropriate acquisition parameters were strictly adhered to during the imaging procedure; and (d) individuals’ age ranged from 18 to 70 years. Exclusion criteria were applied to the patients’ medical records according to the following parameters: (a) presence of fractures, significant bone alterations in the jaws, or craniofacial asymmetries; (b) a prior history of osteosynthesis, orthognathic surgery, or antineoplastic treatment during childhood that could potentially affect the development of the maxillofacial area, particularly the dentition; (c) congenital or acquired diseases or syndromes that may have influenced the structure or morphology of the jaws and teeth; (d) the presence of radiographic splints or plaques associated with the mandible during CBCT imaging; and (e) imaging distortions, such as artifacts or low-quality CBCT images. In addition, medical records were thoroughly reviewed in order to obtain information on the sex, date of birth, and date of the respective CBCT scan of each patient.

Each patient’s volumetric data were stored in Digital Imaging and Communications in Medicine (DICOM) format. These data were reprocessed using ViewBox 4 software (version 4.1.4.1; dHAL Software, Kifissia, Greece; www.dhal.com, accessed on 28 January 2025), which allowed the reconstruction of cross-sectional images and the creation of new images. The complete volume was visualized in all three vertical axes (x-, y-, and z-axes).

**Figure 1:**
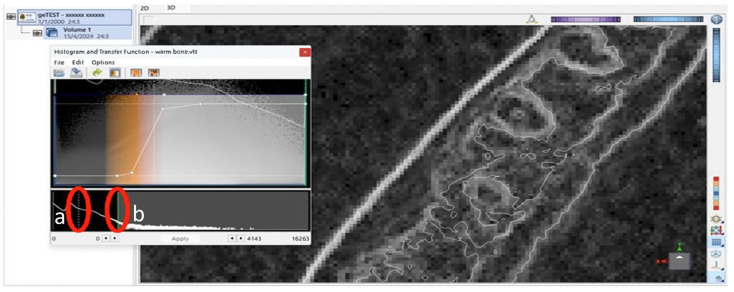
Screenshot of the software interface showing the histogram and transfer function editor. Two vertical markers were displayed: (**a**) a thin white dotted line, indicating a reference point along the grayscale intensity axis, and (**b**) a solid green line, representing the threshold value applied for segmentation or visualization adjustment.

To provide accurate delineation of the external surfaces, a thin dotted line was precisely positioned at the center of the external buccal and lingual surfaces of the mandible, aiming to more accurately represent its morphology, as illustrated in the axial image. As shown in Figure 1, the green’s line placement also aimed to segment and/or adjust the image’s visualization.

**Figure 2:**
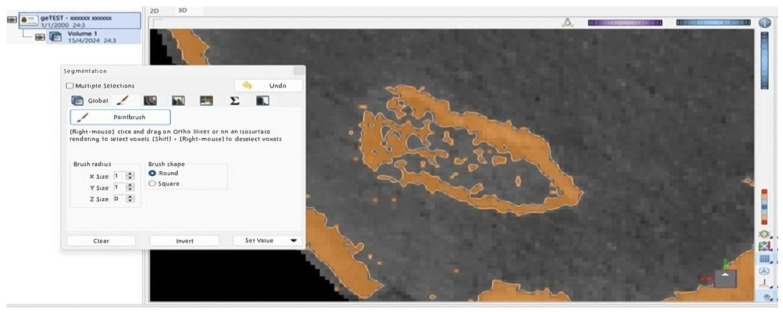
Brown-shaded area of the right condyle’s surface is identified as bone by the ViewBox 4 software. Discontinuity was observed on the external surface of the condyle.

**Figure 3:**
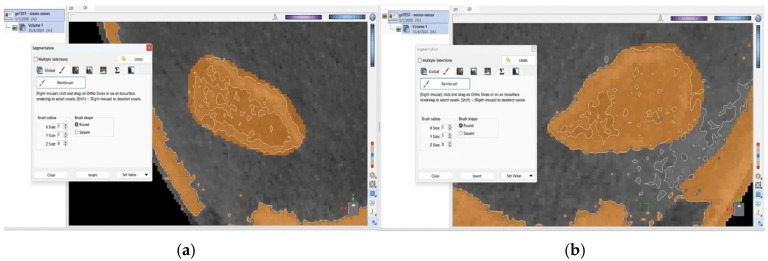
External, brown-shaded surfaces of the (**a**) right and (**b**) left condyle following manual correction.

In the exceptionally unusual event that an omission or irregularity occurred during this processing of the image, each observer was able to manually define the precise boundaries for the mandibular bone across each cross-section along the *z*-axis. This step represented a fundamental cornerstone, as it was essential to isolate its volume from adjacent anatomical structures, such as the temporal bone, before proceeding with further analysis (Figure 2 and Figure 3).

**Figure 4:**
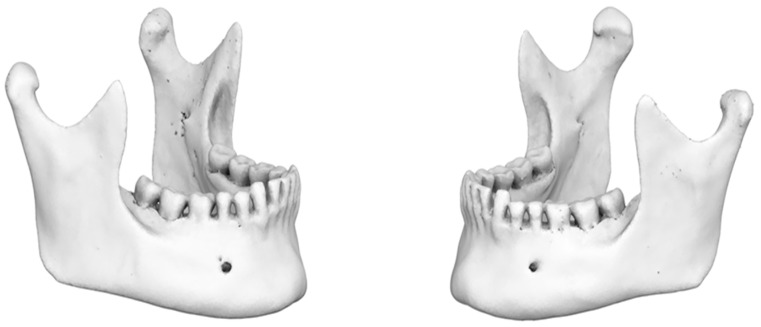
Three-dimensional mandibular reconstruction (coronal plane).

Upon completion of the previous procedure, a three-dimensional surface reconstruction of the skull was performed. The volume of the surrounding skull, excluding the region of interest (the mandible), was filtered and removed using specific parameters implemented in ViewBox 4 software (Figure 4).

Subsequently, each observer manually performed accurate placement of markers on specific anatomical points of the mandible, which were defined as ‘initial digitized points’ in the ViewBox 4 software.

**Figure 5:**
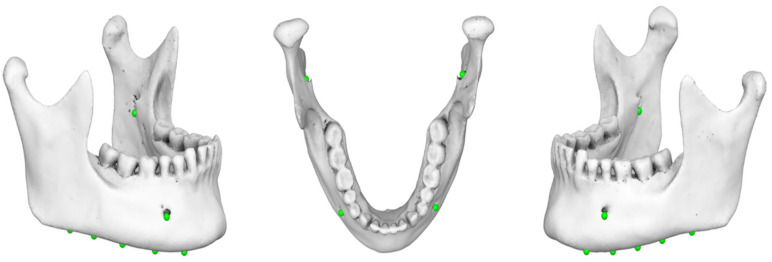
Three-dimensional mandibular reconstruction in which the ‘initial digitized points’ have been accurately identified in the axial and sagittal planes.

The four ‘digitized points’ were as follows:

i. inferior point of right mental foramen (Mental Foramen R),
ii. inferior point of left mental foramen (Mental Foramen L),
iii. inferior point of right mandibular foramen (Mand Foramen R),
iv. inferior point of left mandibular foramen (Mand Foramen L) (Figure 5).

**Figure 6:**
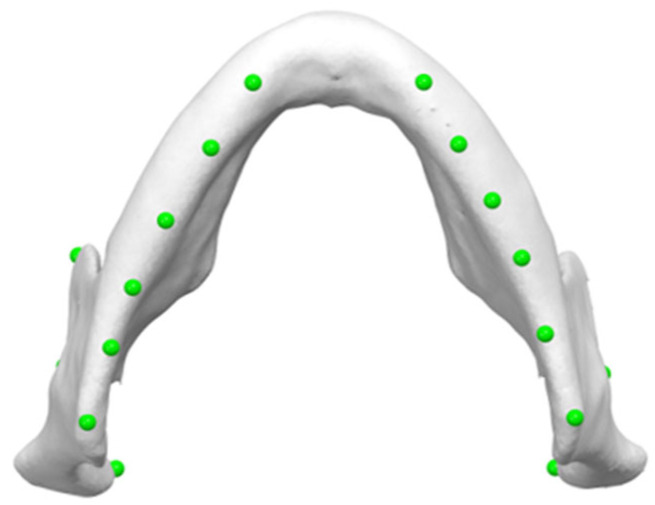
Ten points bilaterally placed along the inferior border of the mandible, serving as markers for determining mandibular reference plane (Mand Border points).

The reference plane was then established by labeling ten points bilaterally along the inferior border of the mandible (Figure 6). These points were chosen to ensure greater accuracy, as they are all located along the inferior border of the mandible. Then, the software automatically generated a reference plane using a best-fit approach. The mandibular reference plane was established by aligning the central points of each marker to ensure that they were all located in the same plane.

**Figure 7:**
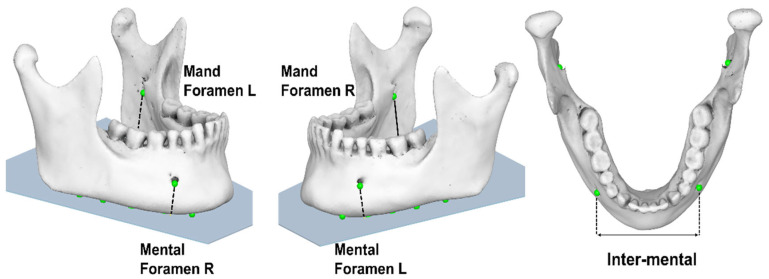
Linear measurements (‘Inter-mental’, ‘Mental Foramen (right/left)’, ‘Mand Foramen (right/left)’ obtained from 3D CBCT reconstruction.

Specifically, the projections of the ‘initial digitized points’ onto the mandibular reference plane were defined as follows:

i. projection of the inferior point of (R) mental foramen (‘Mental Foramen R proj’),
ii. projection of the inferior point of (L) mental foramen (‘Mental Foramen L proj’),
iii. projection of the inferior point of (R) mandibular foramen (‘Mand Foramen R proj’),
iv. projection of the inferior point of (L) mandibular foramen (‘Mand Foramen L proj’),
v. estimated linear measurement between the projections of inferior points of mental foramina (at the reference plane) (Inter-mental) (Figure 7).

The recorded measurement values were documented in a Microsoft© Excel© 365 spreadsheet (version 16.80; Microsoft Corp., Redmond, WA, USA), and this procedure was repeated for each subsequent patient.

All sex estimation measurements were performed under identical conditions, blinded to the sex, following the prior standardization procedure. Both observers were experienced oral and maxillofacial radiologists.

To assess measurement reliability, both intra- and inter-observer reliability were evaluated. One month later, both observers repeated the measurements. A third observer, an experienced oral and maxillofacial radiologist, was consulted in case of any discrepancy. Subsequently, patients were classified into four age-based groups as follows:

- 1st group: 18–29 years old
- 2nd group: 30–40 years old
- 3rd group: 41–51 years old
- 4th group: 52–70 years old

The selected age range (18–70 years) was based on both developmental and anatomical considerations. The lower age limit of 18 years ensured the skeletal maturation of the included individuals. It is evident that after 70 years, morphological changes may have occurred in the mandible that might affect the accuracy of sex-related measurements. Specifically, Goyushov et al. (2020) reported that mandibular morphology may become increasingly variable beyond the age of 70, potentially compromising the consistency of sex estimation models. This variability is based on the persistent cortical bone remodeling associated with aging [15]. Moreover, according to Ishibashi et al. (1995), severe degenerative changes (such as erosive lesions, flattened or polygonal cortical bone shapes) modify the structural integrity of the condyle and may reduce the reliability of morphometric measurements in individuals older than 70 years [16].

The measurement results were documented in tables and subjected to statistical analysis using the Statistical Package for the Social Sciences (SPSS^®^ v. 24, IBM Corp., New York, NY, USA), with a significance level set at *p*-value ≤ 0.05.

## 3. Statistical Analysis

Demographic variables and the distribution of inter-mental and all linear measurements (in mm) were examined with respect to mandibular foramina regions. The analysis comprised two distinct phases. In the initial phase of univariate analysis, independent *t*-tests were applied to assess mean differences by sex across all measurements. Pearson correlation coefficients were evaluated to investigate potential correlations among the variables. Considering the statistically significant findings from the first stage, a multivariate binary logistic regression (BLR) model was developed in the second stage. Prior to the development of the BLR model, the following assumptions were verified: (1) absence of outliers; (2) absence of multicollinearities among the independent variables (measurements); and (3) that there was a linear relationship between the independent variables and the logit of the dependent variable (sex). Pseudo-R^2^ statistics were applied to evaluate the quality of the univariate and multivariate models. Furthermore, an accuracy analysis was performed by evaluating a dataset consisting of a training (80%) and a testing sample (20%). A stratified random sample technique was implemented for selecting the training and testing subsets. This procedure provided the reduction of selection bias across the sex and age groups. The receiver operating characteristic curve (ROC curve) was implemented to validate the model’s prediction performance; *p*-values < 0.05 were considered as statistically significant.

## 4. Results

The mean age (± standard deviation (SD)) of the patients was 50.7 ± 13.4 years, the minimum and maximum ages were 18 and 70 years, respectively. There was no statistically significant difference in the age distribution between males and females (*p*-value = 0.626). Table 1 displays the sample’s distribution of age and sex.

The descriptive statistics of measurements (mean differences between females and males participants) are presented in Table 2. Both males and females exhibited sexual dimorphism, as evidenced by consistently higher values in males across the measured variables: i. “Inter-mental”, ii. “Mental Foramen R”, iii. “Mental Foramen L”, iv. “Mand Foramen R”, and v. “Mand Foramen L”. Using *t*-test analysis, statistical significance was verified (*p*-value < 0.001).

Intra- and inter-rater reliability were estimated using the intraclass correlation coefficient (ICC), yielding values of 0.91 and 0.88, respectively, indicating excellent and good agreement.

Pearson correlation coefficients (r) for all measurements are presented in Table 3. The “Inter-mental” distance demonstrated moderate correlation with “Mand Foramen R” and “Mand Foramen L” linear measurements. Hence, moderate associations were also evident between “Mand Foramen R”/“Mand Foramen L” and “Mental Foramen R”/“Mental Foramen L”. Within the accepted boundaries (r = 0.60–0.79), these coefficients indicate a moderate to strong linear relationship, whereby increases in one linear measurement were consistently associated with proportional increases in the other.

All associations were statistically significant (*p*-value < 0.05) except for those involving the variables “Inter-mental”, “Mental Foramen R” and “Mental Foramen L”.

Table 4 presents the model fit statistics of univariate binary logistic regression models, which were applied to identify the most predictive measurements for sex estimation. The highest pseudo R^2^ values are marked in bold.

**Figure 8:**
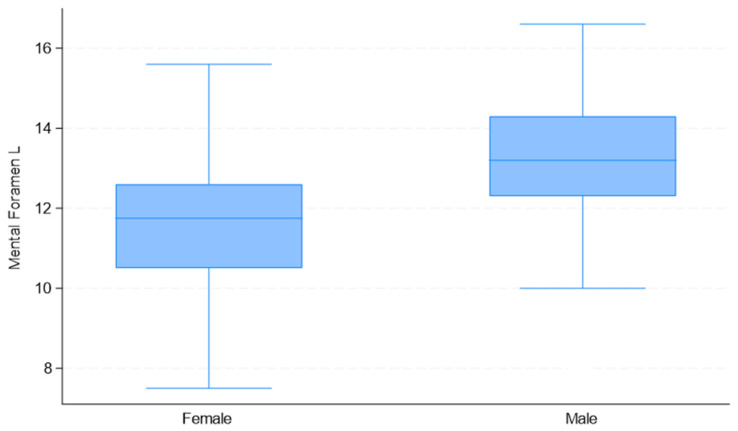
Box plots of “Mental Foramen L” distribution used in the development of the multivariate binary logistic regression model, by sex.

**Figure 9:**
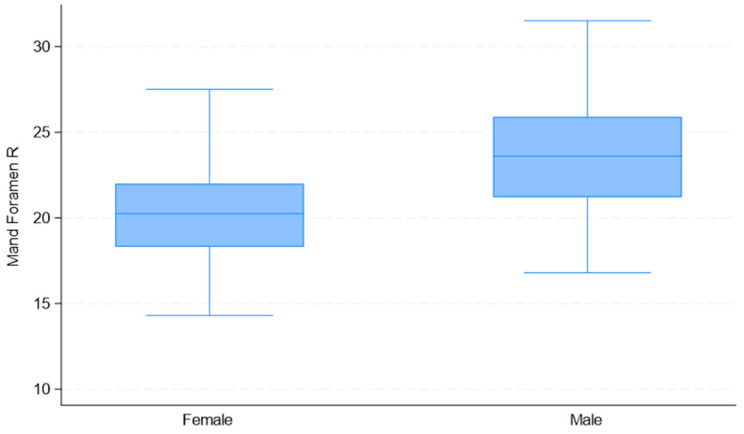
Box plots of “Mand Foramen R” distributions used in the development of the multivariate binary logistic regression model, by sex.

**Figure 10:**
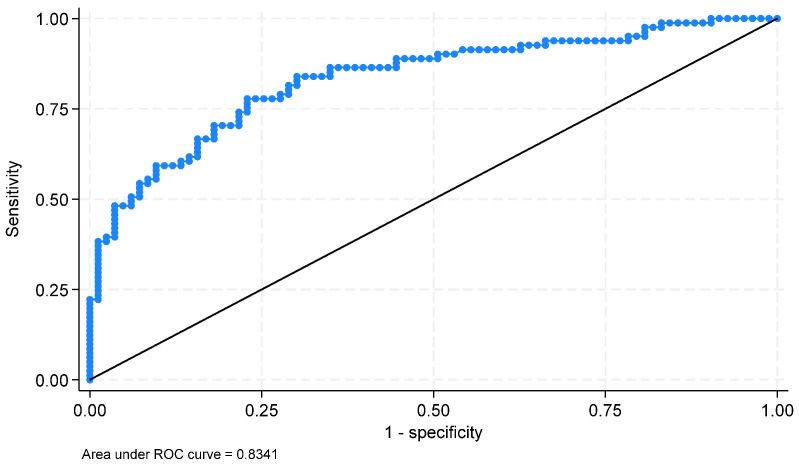
Receiver operating characteristic curve (ROC curve) of the diagnostic capacity of the developed model.

Upon considering the observed measurement differences between males and females (Table 2), the correlations among variables (Table 3), and the quality of univariate models (Table 4), the selected predictor variables for sex estimation were “Mental Foramen L” and “Mand Foramen R”. The distribution of these measurements by sex is presented in Figure 8, Figure 9 and Figure 10.

The estimated equation of the BLR model is shown in Table 5. The generated model achieved an accuracy of 66.7% in sex prediction. In the training sample, sex was estimated with an accuracy of 49.5%. The observed discrepancy may be attributed to model instability, which can occur in studies with limited sample sizes and potential class imbalance. To increase accuracy, additional metrics were performed, such as the area under the ROC curve (0.834), sensitivity, and specificity (76.5% and 77.1%), indicating that the model performs well.

To recognize a female participant, a value lower than 12.6 mm and 22 mm in “Mental Foramen L” and “Mand Foramen R”, respectively, can be used. Overall, for the BLR model, a value lower than 1.15 recognizes a female participant, and a value higher than 1.15 a male participant.

The estimated sensitivity and specificity were 76.5% and 77.1%, respectively, indicating that the model had very good adjustment. Figure 10 presents the ROC curve of the diagnostic capacity of the developed model. The area under the curve (AUC) is 0.834 with a 95% confidence interval: (0.769 to 0.899); 76.8% of the subjects were correctly classified.

## 5. Discussion

Sex estimation is regarded as a pivotal element in the identification process and also a highly valuable component of forensic anthropology and medicolegal investigations [17,18].

Several identification methods have been studied by forensic scientists to determine the sex of a skeletal specimen, which include molecular, morphological, and morphometrical techniques [19]. Molecular analysis of DNA has been identified as a difficult procedure due to the sample’s degradation and contamination [20]. In contrast, morphological features’ interpretation is characterized by subjectivity and presents a greater probability of inaccuracy, while morphometric methods are considered fast, simple, and cost-effective [21].

A limited number of studies conducted in Egypt, Japan, and Brazil have investigated sexual dimorphism by evaluating mandibular osteometric measurements (relative location of the anatomical foramina, such as mandibular foramen, mental foramen (MF), and the mandibular canal (MC)) and have reported discrepancies in their populations [22,23,24,25,26]. The study of adult sexual dimorphism is enhanced by hormonal and endocrine stimuli that modulate sex differentiation [27].

In our study, the estimated projected linear measurement of the most inferior point of the mental foramen (bilaterally) [“Dist Mental Foramen (R and L)” variable] to the mandibular reference level of the mandible was assessed as statistically significant between sexes (*p*-value < 0.001 *).

In a recent study by Chanda et al. (2023), the ILM parameter (defined as the distance from the most inferior point of the mental foramen to the most inferior border of mandible), measured on cross-sectional views of CBCT scans, had statistically significant differences between sexes (*p*-value < 0.0001) [19]. The authors also concluded that it was feasible to determine sex through assessment of the mental foramen’s morphometrics on CBCT images, with a maximum predictive value of up to 89% in the Indian population. This specific outcome provided clear evidence of sexual dimorphism, as all males’ linear measurements were greater in average than females’ [19]. These results were consistent with the corresponding outcomes of the study conducted by Subash et al. (2019) in a South Indian population [28]. According to studies conducted on various populations, including the Polish [29], Egyptian [30], Turkish [31], and Jordanian [32] populations, comparable results were documented. On the contrary, a study in the Iranian population reported no sexual dimorphism concerning mental foramen measurements (vertical plane) [33]. The authors hypothesized that these discrepancies may be attributed to inherent racial diversity and different sample sizes. A recent study on Peruvian volunteers also reported a statistically significant sex difference in the vertical position of the mental foramen (*p*-value < 0.001). The mean value of this measurement was estimated at 13.86 mm on average and showed significant side variation. The researchers identified that the mental foramen was located closer to the mandibular border in females than in males [34]. Similarly, Von Arx et al. (2013) reported the same distance, averaging at 13.2 mm with no lateral differences but with significant sexual dimorphism [35]. Kalender et al. (2012) demonstrated an even lower measurement at 12.4 mm with no relevant lateral differences, but males showed greater estimated measurements compared to females [36]. The consistently greater distance in males reported by these studies has been linked to the smaller mandible’s size found in females [26,37].

According to the study by Ahmed et al. (2024), the same predictor for sex estimation (measured in cross-sectional planes) did not reveal any statistical significance (*p*-value > 0.05) [38].

In our study, the bimental foramina breadth parameter (“Inter-mental” variable), defined as the linear measurement (distance) of the projections of the mental foramina’s inferior points onto the mandibular reference plane, demonstrated statistically significant differences between the sexes (*p*-value < 0.001 *).

Similarly, in the study by Lenin et al. (2024), the bimental foramina breadth parameter also showed statistically significant differences between sexes (*p*-value < 0.001) by evaluating 3D reconstruction images obtained from DICOM files [39]. Comparable outcomes were reported by Saini et al. (2022) and by Dong et al. (2015) (*p*-value = 0.009 and *p*-value = 0.000, respectively) [40,41].

Deng et al. (2017) evaluated 219 CBCT images (adult population) obtained from the Department of Oral Radiology (School of Stomatology, Wuhan University, People′s Republic of China). All participants were of Han ethnicity and lived in Hubei Province, central China. Their sample consisted of individuals without mandibular deformation, fracture, previous surgical history in the mandible region, severe osteoporosis on CBCT images, or metabolic–endocrinological diseases. Statistically significant sexual dimorphism was also observed in the bimental foramina breadth parameter (*p*-value = 0.000), exhibiting a low accuracy for sex estimation of 65.3% [42]. Their results were also consistent with those of other studies [31,43].

In the recent study by Machado et al. (2024), the MFD parameter (distance between the mental foramina) was statistically significant for predicting sex (*p*-value = 0.021). In this study, they assessed linear measurements among the most superior points of mental foramina by evaluating 3D reconstructions of CBCT scans (stored in DICOM files) and thus proceeded to statistical analysis. The accuracy rate was greater than 80% regarding males and above 82% for females [44].

In our study, the projected linear distance between the inferior point of the mandibular foramen (“Dist Mand Foramen (R and L)” variable) to the mandibular reference level demonstrated statistically significant sexual dimorphism (*p*-value < 0.001 *).

In the study by Rath et al. (2022), a total of 120 CBCT images (1:1 male to female ratio) of adult dentate individuals aged between 18 and 60 years were analyzed to identify sexual dimorphism in an eastern Indian population based on the mandibular foramen’s position. Ten linear measurements (8 on coronal slices and 2 on axial slices) were analyzed for the study. The PMaF parameter (linear measurement between the most inferior point of the mandibular foramen to the lowest mandibular point) assessed in axial planes showed significant differences between sexes (*p*-value < 0.0001), with all mean values recorded as greater in males than females [45].

Rashid et al. (2024) evaluated mandibular foramen’s position in 3D reconstructed panoramic images from cone beam computed tomography (CBCT) records. The sample consisted of 56 dentate participants (22 males, 34 females) aged between 20 and 55 years. Statistical analysis, such as *t*-test and ANOVA, was performed to evaluate linear measurements of mandibular foramen at specific anatomical landmarks regarding sex and age estimation. The authors reported statistically significant sex difference in the linear measurement from mandibular foramen to the posterior border of the ramus (*p*-value < 0.0001) [46]. Correa et al. (2019) reported opposite outcomes, by investigating the mandibular foramen’s position in terms of different facial shapes [47]. In addition, Park and Lee (2015) investigated the mandibular foramen’s location in patients with different types of malocclusion and demonstrated non-significant sex-related differences in patients with normal occlusion [48].

Amin et al. (2018) and Le et al. (2024) investigated the potential sexual dimorphism of the mandibular foramen’s measurements in 3D reconstructed CBCT images. Both reported statistically significant differences between the sexes when measuring from the mandibular foramen’s center to the inferior margin of the mandibular ramus and to the most superior point of the curvature of the sigmoid notch, respectively [32,49]. Ahn et al. (2020) reported similar results. In their study, there was also a statistically significant difference between the sexes when measuring the distance from the center of the mandibular foramen to the most superior point of the curvature of the sigmoid notch [50].

Sexual dimorphism in specific anthropometric parameters of the mandibular foramen was statistically evaluated in a Brazilian population based on 3D reconstructed CBCT scans. The researchers assessed eight linear measurements. Specifically, two linear measurements regarding the linear distance of the MF to (a) the most anterior part of the mandibular ramus, and (b) the most posterior part of the ramus, demonstrated statistical significance between the sexes [25]. Similarly, MDCT studies conducted in Taiwan also reported statistically significant sexual dimorphism of both the mandibular and mental foraminal location [49,50]. On the contrary, Afsar et al. (1998) identified that the mandibular foramen was highly dimorphic, with no sensitivity to age or sex estimation [23].

Dimorphic patterns were investigated in a sample of 190 adult Bulgarians (98 males and 92 females). A total of 45 anatomical landmarks were digitized on 3D surface models of the mandible, and both shape and size were analyzed in relation to sex and age. The study reported considerable differences in mandibular size between sexes, with a classification accuracy of 87.4% using centroid size alone. Shape-based categorization achieved 77.9% accuracy when Procrustes coordinates were utilized, but accuracy decreased to 53.2% when allometric effects were removed, applying regression residuals. Their findings revealed that mandibular size, compared to mandibular shape, was a more accurate sex determination [51,52,53].

A recent study by Japanese researchers evaluated postmortem computed tomography (CT) scans of 200 mandibles from Japanese cadavers for the purpose of determining sex. The study reported a substantial sex-related variance regarding the position of the mental foramen (*p*-value < 0.0001) and an association between age and mental foramen location for both sexes [54]. Significant sexual dimorphism in mental foramen measurements has been reported across various populations. Specifically, the inter-mental foramen distance differed significantly between males and females in South Indian and Chilean samples [39,55], while all linear measurements related to mental foramen location showed similar patterns in a Dutch population [56]. The identical results were observed in an Iranian population, where the mental foramen position demonstrated sexual dimorphism variance [57]. Moreover, morphometric examination of mandibles utilizing cone-beam computed tomography (CBCT) in a South African and in a Brazilian population, correspondingly, revealed that specific linear measurements of the mandibular foramen indicate substantial sexual dimorphism [58,59].

Recent research evidence indicates that the expression of sexual dimorphism in mandibular morphology is significantly influenced by genetic, hormonal, and environmental factors, which vary across populations. According to Ceballos et al. (2025), these biological factors can have a direct impact on bone growth and structure, contributing to population-dependent variability in sex estimation accuracy by applying mandibular measurements in CBCT images [55].

In the study by Mostafa and Abou El Fotouh (2020), similar results were observed, demonstrating that several hormonal and genetic factors might impact the mandibular development. The aforementioned factors are population-specific parameters that influence final mandibular shape and size as well as sex estimation methods [60].

Moreover, external factors such as hormonal alterations, masticatory muscle activity, dietary habits, and environmental conditions may have a major effect on mandibular morphology. These features, which are particularly evident throughout aging and in diverse cultural and dietary circumstances, might influence mandibular shape and size, resulting in inter-population deviations in mandibular morphometrics, and therefore affecting the accuracy of sex estimate approaches [40,61].

In the Greek population, limited studies have explored the implementation of mandibular morphology for sex determination. A geometric morphometric method was recently developed by Chalazoniti et al. (2024) for evaluating 100 CBCT images from Greek individuals’ (50 males and 50 females), achieving a 91% classification accuracy by principal component analysis in form space [11]. Bertsatos et al. (2019) used this approach on a larger sample of 194 dry mandibles from the Athens Collection. For their purpose, 23 linear and angular measurements assessed from 3D surface models and a cross-validated analysis exhibited an accuracy of up to 85.7%. According to their results, the most discriminant features involved coronoid height, ramus height, and mandibular length [12]. Another research investigation combined discriminant function analysis and five typical osteometric variables to perform a morphometric assessment on 70 dry mandibles from the currently available Cretan skeletal collection. The most sexually dimorphic characteristics were identified to be bigonial and bicondylar breadths, and the authors reported an overall classification accuracy of 80% [13].

The localization of specific anatomical landmarks of the mandible in 3D reconstructed CBCT images, as well as the corresponding linear measurements between mental and mandibular foramina, could be highly beneficial for identifying individuals in mass disasters, medico-legal investigations of single cases, and the discovery of skeletal remains during archaeological or anthropological excavations.

At this stage, the results are considered statistically significant for the sample used in the present study. To enhance the predictive performance of the developed BLR equation, we intend to increase the sample size and include additional anthropometric measurements of the posterior mandibular region. This novel approach may enable the development of a new BLR equation, appropriate for sex determination in the general population. The current study’s findings suggest that mandibular linear measurements obtained from CBCT scans may be beneficial for forensic investigations, notably sex assessment in modern Greek adults.

Several anatomical and technical factors may have an impact on the accuracy of CBCT-based mandibular measurements for sex estimation. Edentulism has been linked to alteration of ramus height, which may impact landmark identification and measurement accuracy [62]. Similarly, occlusion type has been demonstrated to influence mandibular morphology. Along with occlusal, demographic factors contribute to intrapopulation morphological variation [63]. In addition, voxel size variability in CBCT scans can impact spatial resolution and image quality. Larger voxel sizes may limit the clarity of anatomical structures due to lowered sharpness and partial volume effects, resulting in measurement accuracy reduction. Consequently, standardized exposure protocols are essential for efficient morphometric analysis in forensic cases [64].

## 6. Conclusions

Males exhibited greater mean values for all anthropometric linear measurements than females. Additionally, linear measurements of specific anatomical landmarks presented a statistically significant difference between sexes in the univariate analysis (*p*-value < 0.001). Applying a binary logistic regression analysis, only two features, the projections of the inferior points of (a) the right mandibular foramen and (b) the left mental foramen, to the mandibular reference plane, can contribute to sex determination with a successful prediction rate of 0.667. According to the developed BLR model, a score less than 1.15 indicates a female, and a score above 1.15 indicates a male individual. In the current study, there was a sex-related dimorphism associated with the relative position of the mandibular and mental foramina, as determined by linear measurements from anatomical reference points in this adult Greek sample. Future large-scale research studies combining additional parameters might assist in developing a forensic database for specific populations and enabling comparison with other ethnicities.

## Figures and Tables

**Table 1:** Sample distribution (*n*; %) of sex and mean (± standard deviation (SD)) age of the 204 patients.

Characteristic	All	Female	Male	*p*-Value
**Sex**	204	102 (50%)	102 (50%)	
**Age (years)**	50.7 (13.4)	50.2 (13.5)	51.1 (13.3)	0.626

**Table 2:** Descriptive statistics of measurements (102 females and 102 males) and mean differences by applying *t*-test.

Distance (mm)	Sex	Mean (SD)	Mean Difference	95% CI	*p*-Value
Inter-mental	Female	46.1 (2.82)	−1.3	(−2.1 to −0.6)	<0.001 *
	Male	47.4 (2.44)			
Mental Foramen R	Female	11.6 (1.51)	−1.4	(−1.8 to −1.0)	<0.001 *
	Male	13.0 (1.64)			
Mental Foramen L	Female	11.7 (1.42)	−1.5	(−2.0 to −1.1)	<0.001 *
	Male	13.2 (1.46)			
Mand Foramen R	Female	20.1 (2.88)	−3.3	(−4.2 to −2.5)	<0.001 *
	Male	23.4 (3.25)			
Mand Foramen L	Female	20.3 (3.41)	−3.1	(−4.0 to −2.2)	<0.001 *
	Male	23.4 (3.28)			

SD: standard deviation; * statistically significant according to the *t*-test (*p*-value < 0.001).

**Table 3:** Correlation matrix of linear measurements.

Measurements	Pearson Correlation Coefficient				
Distance (mm)	Inter-Mental	Mental Foramen R	Mental Foramen L	Mand Foramen R	Mand Foramen L
**Inter-mental**	1				
**Mental Foramen R**	0.05	1			
**Mental Foramen L**	0.12	0.80 *	1		
**Mand Foramen R**	0.23 *	0.36 *	0.33 *	1	
**Mand Foramen L**	0.19 *	0.37 *	0.37 *	0.79 *	1

* statistically significant result (*p*-value < 0.05).

**Table 4:** Results from univariate binary logistic regression models (dependent variable: sex, 0 = female and 1 = male).

Measurement, Distance (mm)	Constant	Slope	Pseudo R^2^
**Inter-mental**	−9.1	0.19	0.045
**Mental Foramen R**	−8.2	0.66	**0.150**
**Mental Foramen L**	−7.7	0.62	**0.154**
**Mand Foramen R**	−7.9	0.36	**0.187**
**Mand Foramen L**	−6.1	0.28	**0.141**

**Table 5:** Binary logistic regression (BLR) model equation with corresponding evaluation metrics.

**BLR equation**	**Log(Y) = −13.15 + 0.499 × Mental Foramen L + 0.319 × M and Foramen R**
**Evaluation parameters**	
Pseudo R^2^	0.667
Accuracy & 95%Confidence Interval	49.5% (45.5% to 53.5%)

## Data Availability

The data that support the findings of this study are available from the corresponding author upon reasonable request.
